# A novel *CACNA1E* mutation (c.1256G > A) was identified in a Chinese patient with epilepsy and congenital heart disease

**DOI:** 10.3389/fmed.2026.1865188

**Published:** 2026-07-21

**Authors:** Juan Pan, Mei-Fang Zhao, Zhao-Chuan Liu, Ruo-Bing Li, Jie-Yuan Jin, Can-Bo Tang, Zhen-Bo Cheng

**Affiliations:** 1 Hunan Provincial People's Hospital, The First Affiliated Hospital of Hunan Normal University, Hunan Normal University, Changsha, Hunan, China; 2 School of Life Sciences, Central South University, Changsha, China; 3 Department of Clinical Medicine, Xinjiang Medical University, Urumqi, China; 4 School of Medicine, Shaoxing University, Shaoxing, China; 5 People's Hospital of Ningxiang City, Hunan University of Chinese Medicine, Changsha, Hunan, China

**Keywords:** *CACNA1E*, congenital heart disease, epilepsy, mutation, whole-exome sequencing

## Abstract

**Background:**

*CACNA1E* mutations cause developmental and epileptic encephalopathy. We report a Chinese pediatric patient with epilepsy and congenital heart disease who carries a novel CACNA1E variant.

**Methods:**

Whole-exome sequencing (WES) was performed on the proband and his unaffected parents. The variant was validated by Sanger sequencing and screened in 200 healthy controls. Bioinformatic tools and ACMG criteria were used for pathogenicity assessment.

**Results:**

A *de novo* heterozygous missense variant, NM_000721.4: c.1256G > A (p.R419Q), was identified. It was absent in both parents and in 200 controls. The variant lies in a highly conserved and intolerant region. ACMG classification: likely pathogenic (PS2 + PM2 + PP3). The patient showed marked short-term response to adrenocorticotropic hormone (ACTH).

**Conclusion:**

This novel variant expands the mutational spectrum of CACNA1E and provides a potential treatment clue for ACTH responsiveness in CACNA1E-related spasms.

## 1 Introduction

Epilepsy is a chronic brain disorder characterized by recurrent seizures, which result from abnormal, excessive, and synchronous neuronal discharges in the brain (1, 2). According to current diagnostic criteria, the diagnosis is typically established after a patient has experienced at least two unprovoked seizures occurring more than 24 h apart. As a common neurological condition, epilepsy imposes a significant disease burden worldwide, with an incidence of approximately 61.4 per 100,000 person-years (3). Epidemiological data indicate marked geographical disparities in the prevalence and incidence of epilepsy, with particularly high rates observed in low-income countries with limited healthcare resources, such as Ecuador, Nigeria, and Ethiopia (4). Epilepsy is more common in countries with lower economic status. This variation may be associated with the higher prevalence of risk factors in these regions, including perinatal injuries, central nervous system infections, and traumatic brain injuries. Notably, spontaneous remission may occur in a proportion of epilepsy cases, even in the absence of treatment. In terms of demographic distribution, epilepsy exhibits a slight gender difference, with several studies reporting slightly higher incidence and prevalence rates in males compared to females. The age distribution follows a characteristic bimodal pattern, with the first peak occurring early in life (during infancy and childhood) and a second peak emerging in older adulthood (5). The developmental contribution related to hormonal balance should be mentioned in this context, as pubertal and perimenopausal hormonal changes have been shown to influence seizure susceptibility. Long-term outcomes of childhood epilepsy vary significantly. While some children achieve sustained seizure freedom and can successfully discontinue medication after standardized treatment, others may develop drug-resistant epilepsy, experience persistent seizure activity, and may suffer from severe neurodevelopmental delay or regression (6). Therefore, early precise diagnosis and individualized treatment are crucial for improving long-term prognosis in patients.

The etiology of epilepsy is highly complex, resulting from the interaction between genetic predisposition and environmental factors (7). In recent years, with the widespread application of genetic sequencing technologies, particularly advancements in methods such as whole-exome sequencing (WES), a large number of epilepsy-related pathogenic genes have been successively identified (8–10). Many of these genes encode ion channel proteins, and mutations in them lead to “channelopathies,” which constitute a crucial molecular basis for genetic epilepsies (11). Ion channels play a central role in maintaining neuronal membrane potential stability and regulating the excitation-inhibition balance; dysfunction in these channels can directly lead to abnormal synchronous discharges in neuronal networks, thereby triggering seizures (12, 13). Among the well-established channel genes, mutations in the *SCN1A* gene, which encodes the α1 subunit of the voltage-gated sodium channel, are strongly associated with Dravet syndrome (14). Mutations in the *KCNQ2* gene, encoding the Kv7.2 subunit of the voltage-gated potassium channel, are commonly found in benign familial neonatal epilepsy (15, 16). Furthermore, the *CACNA1A* gene, encoding the α1A subunit of the P/Q-type voltage-gated calcium channel, is linked to childhood absence epilepsy and certain epileptic encephalopathies (17), while the *CACNA1E* gene, encoding the α1E subunit of the R-type calcium channel, has also been implicated in developmental and epileptic encephalopathies (18, 19). Meanwhile, CACNA1E mutations have also been reported to be associated with cardiac disorders, including coronary artery calcification and early repolarization syndrome (20). Of note, the studies reporting these associations were conducted in patient cohorts with confirmed epileptic encephalopathy or developmental delay, rather than in healthy individuals without seizure history; therefore, the findings are specific to affected populations. These discoveries not only reveal that ion channel dysfunction is a common mechanism underlying various epilepsy syndromes but also provide insights for genotype–phenotype correlation.

This study investigated a proband with epilepsy and his parents originating from Hunan, China. The proband in this family presented with clinical manifestations of epilepsy, yet routine examinations failed to identify a clear etiology, thus falling under the category of epilepsy of unknown cause. To elucidate the underlying genetic etiology, we performed WES on peripheral blood DNA samples from the proband and his parents. A series of bioinformatics analyses were then conducted to screen for gene variants associated with the epileptic phenotype, aiming to explore the potential genetic factors of the disease in this patient.

## 2 Materials and methods

### 2.1 Participants and ethical approval

There was a Chinese patient with no previous underlying medical conditions who came to our hospital for examination due to recurrent convulsions at home for over a month. We collected the patient’s medical history and collected peripheral blood from the proband and his biological parents. To exclude common single nucleotide variants (SNVs) that might be present in the general population, we included 200 healthy Han Chinese individuals (100 males, 100 females; age range 2–60 years) without personal or family history of epilepsy or neurodevelopmental disorders. All participants provided peripheral blood samples for subsequent genetic validation. This study was approved by the Ethics Committee of Hunan Provincial People’s Hospital (The First Affiliated Hospital of Hunan Normal University), with the approval number [2025]-185. All participants in this study signed informed consent forms.

### 2.2 Whole-exome sequencing

Genomic DNA was extracted from peripheral blood cells of all participants using the DNeasy Blood & Tissue Kit (Qiagen, Valencia, CA, USA). The WES service and bioinformatics analysis were supported by Novogene Bioinformatics Institute (Beijing, China). Exon capture was performed with the SureSelect Human All Exon V6 Kit (Agilent, Santa Clara, CA, USA), and sequencing was carried out on a HiSeq X-10 system (Illumina, San Diego, CA, USA). Data filtering strategies were designed with reference to published literature and previous findings from our laboratory. The sequencing generated approximately 12 Gb of raw data, with a target region coverage of 97.75 and 99.1% of the target region achieving a sequencing depth of ≥10×. Quality control included removal of adaptor sequences, low-quality bases (Phred score <20), and PCR duplicates.

### 2.3 Mutation validation and cosegregation analysis

Candidate variants identified by WES were validated by Sanger sequencing. Segregation analysis was further performed in the participating family members. Primers were designed using the PrimerQuest Tool from Integrated DNA Technologies. The primer sequences for CACNA1E exon 5 were: forward 5’-GAGAACCGAAGGGCTTTCAT-3′, reverse 5’-AAAGTGCAAAGGTAGACGGG-3′. Subsequent PCR amplicons were sequenced on an ABI 3100 Genetic Analyzer (Applied Biosystems, Foster City, CA, USA).

### 2.4 Bioinformatics analysis

Tolerance, conservation, and mutant modeling analyses were performed using MetaDome,^1^ ConSurf,^2^ and SWISS-MODEL,^3^ respectively. The functional impact of mutations was predicted using MutationTaster.^4^ Intrinsically disordered regions (IDRs) were predicted using the Predictor of Natural Disordered Regions (PONDR; https://www.pondr.com/). The hydrophobicity profile was analyzed using the ProtScale tool on the Expasy server.^5^

## 3 Results

### 3.1 Clinical features

**Figure 1:**
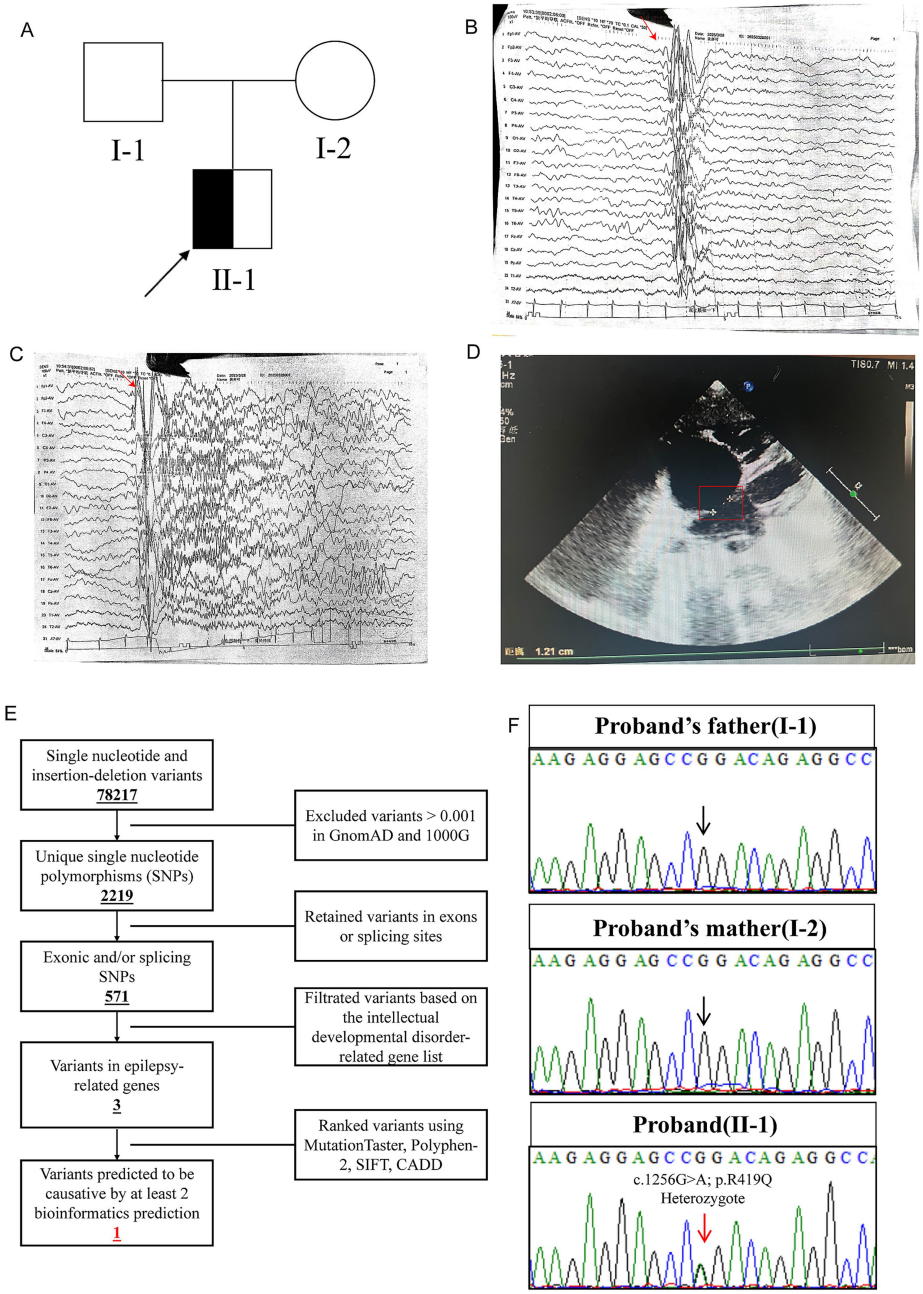
Trio structure of the proband and his parents. **(A)** Pedigree of the proband’s family. Males are denoted by squares, females by circles; affected individuals are indicated by filled symbols, unaffected individuals by open symbols. The arrow points to the proband. **(B,C)** Electroencephalogram (EEG) recordings during sleep. **(B)** A sudden bilateral arm elevation (arrow). **(C)** Head lifting accompanied by sustained tonic activity in all four limbs (arrow). **(D)** Echocardiography revealed an atrial septal defect (box). **(E)** Through an iterative filtering pipeline, candidate mutations in three genes were identified and ultimately narrowed down to a single likely pathogenic variant in one gene. **(F)** Analysis and identification of the *CACNA1E* gene mutation were performed in the proband and his parents. The proband was found to carry a heterozygous missense mutation (c.1256G > A).

The patient is a 2-year-and-9-month-old Chinese boy (Figure 1A) with a previously healthy constitution and no significant medical history. Approximately 1 month ago, the child experienced sudden onset of seizures without obvious cause, with symptoms recurring intermittently over the following month. After admission, frequent nodding movements were observed, which resolved spontaneously. An electroencephalogram (EEG) was performed, revealing generalized epileptiform discharges—including spikes, sharp waves, spike-and-slow waves, sharp-and-slow waves, and sharp slow waves—during both awake and sleep states, with discharges being more prominent during sleep (Figures 1B,C). Throughout the monitoring period, 24 episodes of spasmodic seizures and 3 episodes of tonic spasms were recorded during awake and sleep phases, presenting with typical features and high frequency. In addition, echocardiography revealed an atrial septal defect in the patient (Figure 1D). Following documentation of the patient’s seizure manifestations and EEG findings, peripheral blood samples were collected from the child and both parents for genetic testing.

At the time of genetic testing, the proband was 2 years and 9 months old. No definitive evidence of intellectual disability, developmental regression, or autism spectrum features was yet apparent, although formal developmental assessments were not performed. Long-term follow-up will be necessary to determine whether the p.R419Q variant contributes to neurodevelopmental outcomes beyond epilepsy.

### 3.2 Genetic analysis results

Following alignment and single-nucleotide variant (SNV) calling, 78,217 variants were initially identified in the proband. Through filtration according to the workflow illustrated in Figure 1E, three potentially pathogenic variants were initially prioritized in the proband. Among these, the *CACNA1E* c.1256G > A variant was specifically selected for further validation based on the following criteria: (i) it was a *de novo* heterozygous variant absent in both unaffected parents; (ii) it is located in a highly conserved region of the CACNA1E protein; (iii) it was predicted to be damaging by multiple in silico tools; and (iv) CACNA1E is a well-established epilepsy-associated gene (18, 19). No other potentially pathogenic variant in known epilepsy-related genes was found in the proband. This ultimately led to the identification of a likely pathogenic variant in the *CACNA1E* gene (Table 1).

**Table 1:** The information and pathogenicity classification of the *CACNA1E* variant identified in the proband.

Gene	Variant	Inheritance	Pathogenicity prediction	GnomAD	OMIM clinical phenotype	American college of medical genetics classification
*CACNA1E*	NM_000721.4: c.1256G > A, p.R419Q	*de novo*	MutationTaster: D SIFT: D CADD:24.9 M-CAP: D	0.00001	AD, Developmental and epileptic encephalopathy 69.	Likely pathogenic (PS2 + PM2 + PP3)

D, disease-causing; AD, autosomal dominant.

Validation by Sanger sequencing and co-segregation analysis confirmed a heterozygous *CACNA1E* mutation (NM_000721.4: c.1256G > A; p.R419Q) in the proband, which was absent in both parents (Figure 1F). This mutation was also not detected in our healthy control cohort of 200 individuals. Located in exon 5 of the *CACNA1E* gene, this variant leads to a substitution of arginine (R) to glutamine (Q) at amino acid position 419 of the protein.

**Figure 2:**
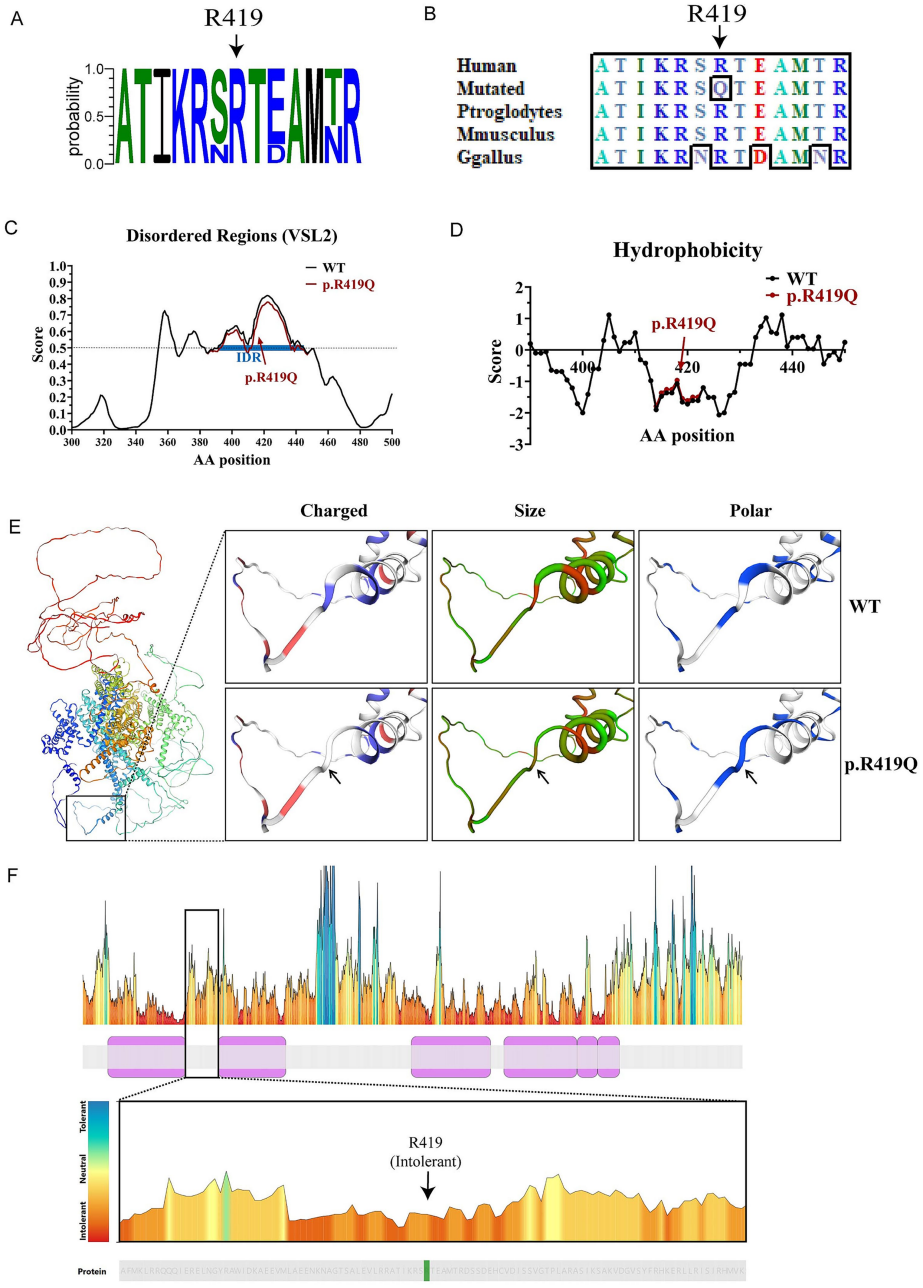
Functional impact analysis of the p.R419Q mutation in the CACNA1E protein. **(A)** Local sequence logo surrounding the conserved residue R419. **(B)** Multiple species conservation analysis of the CACNA1E amino acid sequence. **(C)** Prediction of disordered regions within the protein sequence. **(D)** Analysis comparing the hydrophobicity of the wild type arginine (R419) and the mutant glutamine (Q419). **(E)** Structural modeling showing the local conformational alteration resulting from the p.R419Q substitution. **(F)** Mutational tolerance analysis, indicating that the variant site is located in an intolerant region.

We subsequently performed a series of bioinformatic analyses on this mutation. Conservation analysis using WebLogo and MutationTaster revealed that amino acid position 419 of the CACNA1E protein is highly conserved across species (Figures 2A,B). Prediction of intrinsically disordered regions (IDRs) indicated that the p.R419Q mutation significantly altered the local disorder propensity of the protein sequence (Figure 2C), potentially extending or modifying the structural properties of the IDR. However, hydrophobicity prediction showed minimal difference between the wild-type and mutant proteins at this site (Figure 2D), suggesting that the mutation may not substantially affect the overall hydrophobic character of the protein.

Further protein structure prediction analysis (Figure 2E) demonstrated that the mutation induced changes in charge, side-chain size, and polarity near the variant site, which could affect protein function or interactions with other molecules. Finally, MetaDome analysis predicted that amino acid position 419 resides within a genetically intolerant region of the CACNA1E protein (Figure 2F). In summary, based on the above findings, we propose that the *CACNA1E* p.R419Q mutation is a likely pathogenic variant contributing to the patient’s condition.

We systematically evaluated the variant according to ACMG/AMP guidelines (Table 1). The evidence supported a classification of likely pathogenic: *de novo* occurrence in the proband with both parents wild-type (PS2); absence from population databases (gnomAD, 1,000 Genomes) supports PM2. Additionally, the variant was not detected in 200 in-house healthy controls. Multiple in silico tools predicting a damaging effect (PP3). No functional assay was performed, thus the variant did not meet the criteria for pathogenic without functional evidence (PS3).

## 4 Discussion

This study reports a case of early-onset epileptic encephalopathy in a child caused by a de novo heterozygous missense mutation in the *CACNA1E* gene (c.1256G > A; p.R419Q). The patient presented with typical epileptic spasms at 2 years and 8 months of age, and EEG monitoring detected abnormal discharges. To alleviate spastic symptoms, the child was treated with ACTH. Approximately 1 week after initiating medication, the frequency of spasms significantly reduced, and after 10 days, seizure manifestations such as nodding movements and limb tremors had essentially disappeared, indicating a marked short-term therapeutic effect of ACTH in this case.

Previous case reports and clinical studies have suggested that the response to different antiseizure medications varies in *CACNA1E*-related epilepsy; some children respond well to topiramate, valproic acid, or combination therapy (21, 22). The present case demonstrates that ACTH showed a favorable short-term response in this patient, particularly for spasm-onset cases occurring in infancy. However, this is a single-case observation; larger cohorts and functional studies (e.g., patch-clamp recording of Cav2.3 currents) are required to establish genotype-driven therapeutic stratification.

**Figure 3:**
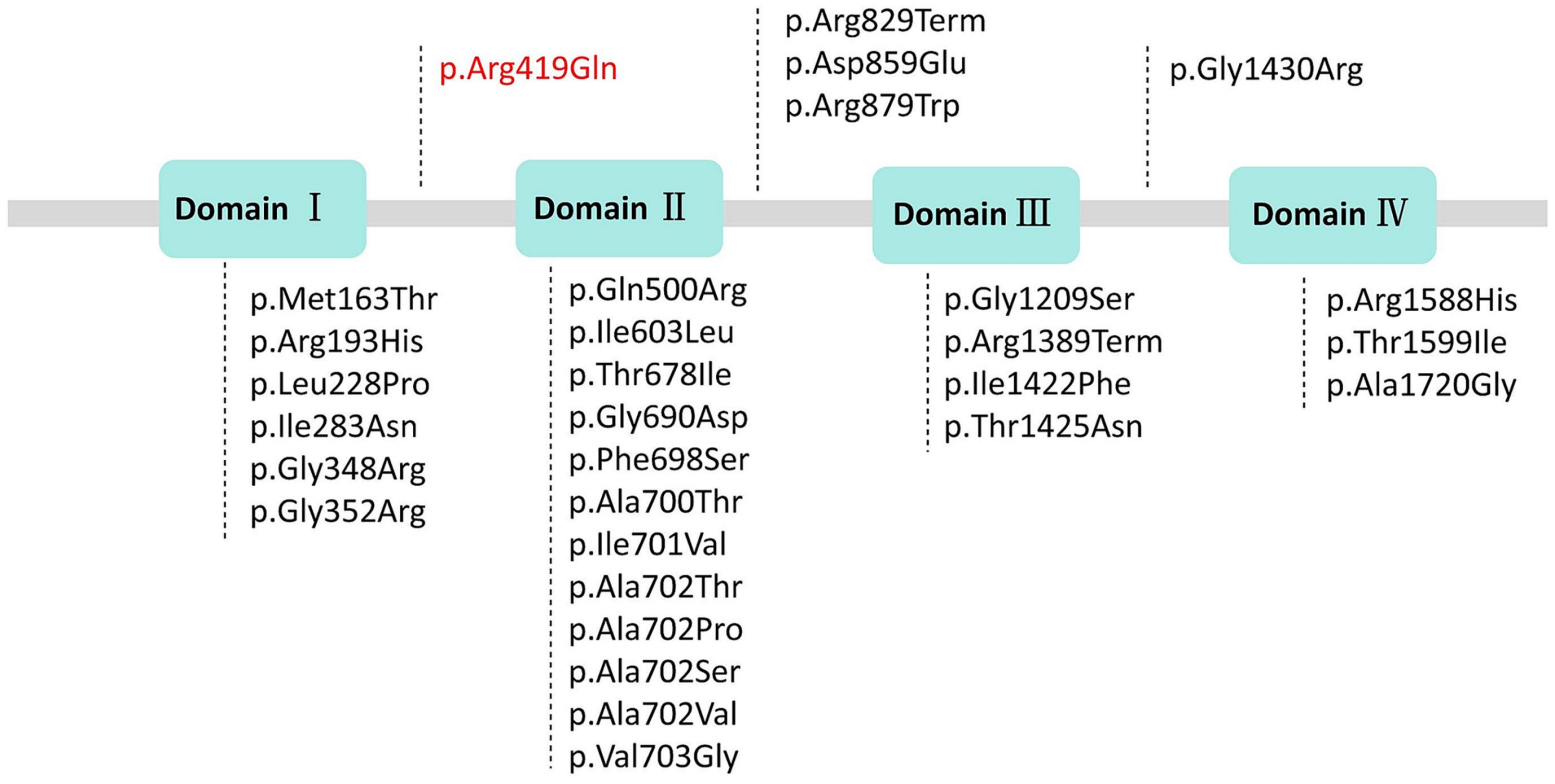
Schematic representation of the CACNA1E protein domains and a summary of reported mutations. The CACNA1E protein is primarily composed of four domains. This diagram summarizes all mutations reported to date. Mutations identified in the present study are highlighted in red.

The CACNA1E gene encodes the alpha 1E subunit (Cav2.3) of the R-type voltage-gated calcium channel, which is widely expressed in the central nervous system and participates in regulating neurotransmitter release and neuronal excitability (23, 24). Here, we summarize the reported mutations of this gene based on their domain locations (18, 25) (Figure 3). The mutation identified in this study is located between domains 1 and 2 of the protein. Although not within a transmembrane region, as part of an intrinsically disordered sequence, it may be involved in interactions with other regulatory molecules. Most pathogenic missense mutations in *CACNA1E* are located within the channel pore region (particularly S4-S6 segments) and typically result in gain-of-function effects. For example, the c.2069G > A (p.Gly690Asp) variant, situated at the cytoplasmic end of the S6 segment in domain II, is strongly associated with refractory infantile epilepsy and severe developmental regression (26). Our variant, p.R419Q, lies in a genetically intolerant region and is predicted to alter local protein structure and disorder propensity, potentially affecting channel function.

It is noteworthy that *CACNA1E* mutations are not invariably associated with epileptic seizures; the phenotypic spectrum also includes intellectual disability, developmental regression, autism spectrum disorder, and even type 2 diabetes and cardiovascular diseases (27, 28). Therefore, in the diagnosis and management of affected patients, attention should extend beyond seizure control to include long-term monitoring of neurodevelopmental and metabolic abnormalities. In the present case, beyond the epileptic spasms and an atrial septal defect detected by echocardiography, no other clinical manifestations such as developmental regression or dyskinesias were clearly identified at the age of 2 years and 9 months. However, given that some CACNA1E-related phenotypes may emerge later in childhood, continuous follow-up is warranted.

This study has several limitations. First, the pathogenicity conclusion is based primarily on in silico predictions, conservation analysis, and genetic evidence, without functional validation (e.g., electrophysiological assessment of channel activity). Specifically, functional studies such as patch-clamp recording are needed to determine the biological effect of the p.R419Q variant on Cav2.3 channel activity. Second, the sample size is a single case with a trio design; no additional affected family members were available. Third, the 200 healthy controls were used only to exclude common polymorphisms and do not provide statistical power for association. Fourth, the ACTH response was observed over a short term; long-term efficacy and safety remain unknown. Future studies should include functional assays and larger patient cohorts to confirm the genotype–phenotype correlation.

## 5 Conclusion

In summary, we identified a heterozygous missense mutation in the *CACNA1E* gene in a Chinese patient with epilepsy. This finding not only provided a diagnostic explanation for the patient’s etiology but also offered valuable information for genetic counseling in the family. Furthermore, it expands the known mutational landscape of the *CACNA1E* gene and provides a preliminary basis for future research in this field.

## Data Availability

The original contributions presented in the study are publicly available. This data can be found here: GSA-Human repository, accession number HRA019749 (https://ngdc.cncb.ac.cn/gsa-human/browse/HRA019749).

## Footnotes

1
https://stuart.radboudumc.nl/metadome/dashboard

2
https://consurf.tau.ac.il/

3
https://swissmodel.expasy.org/interactive

4
https://www.mutationtaster.org/

5
https://web.expasy.org/protscale/
